# Supplementary material for: CD146 Defines a Mesenchymal Stromal Cell Subpopulation with Enhanced Suppressive Properties
Source: Cells. 2022 Jul 22;11(15):2263. doi: 10.3390/cells11152263 (PMC9331786; doi:10.3390/cells11152263)
Supplement: Supplementary file 1 [file cells-11-02263-s001.zip › cells-1779803-supplementary.pdf]

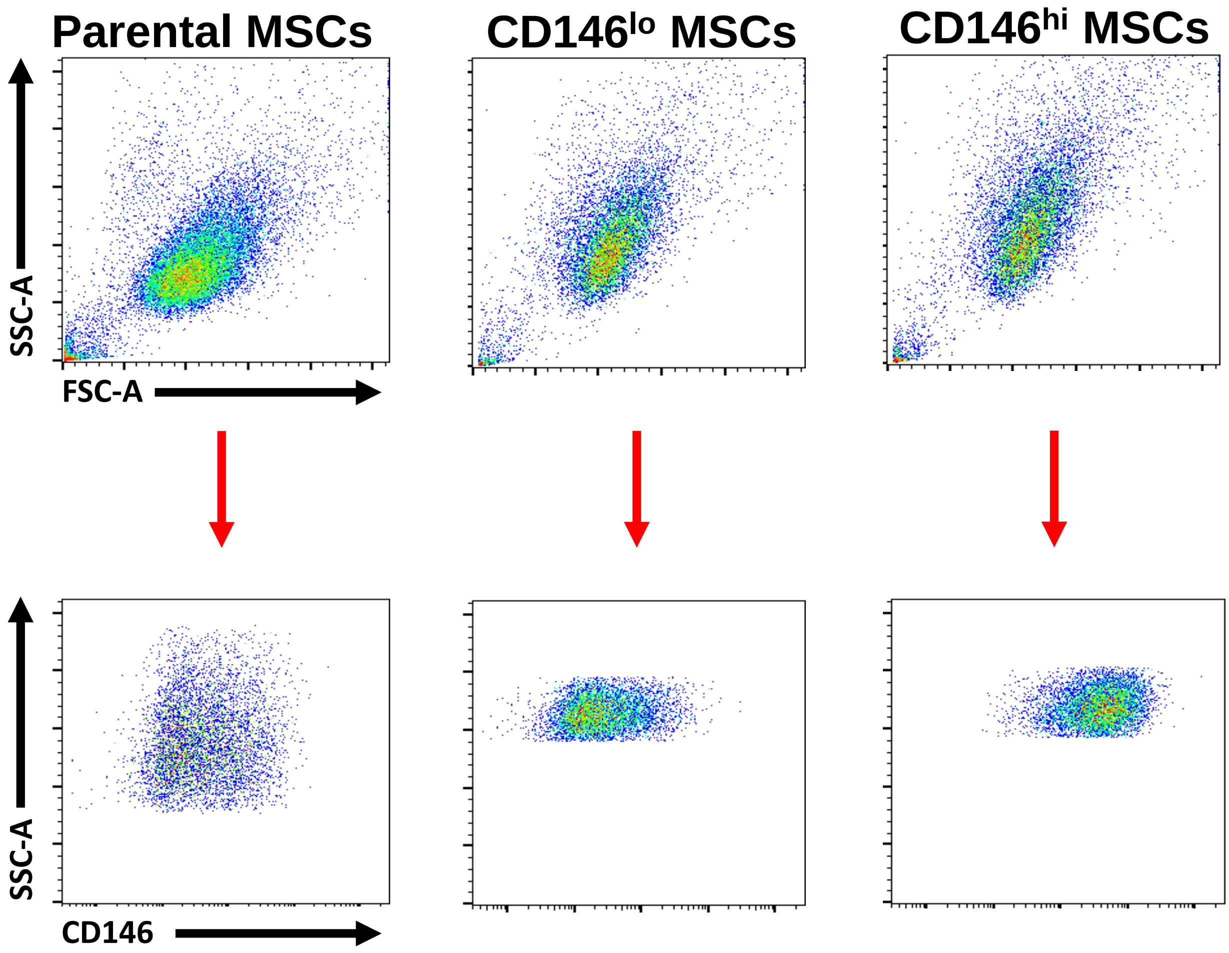

Supplementary figure S1: Dot blots assessment of sorted MSCs. As shown in this panel, both CD146^{lo} and CD146^{hi} MSCs are devoid of any population aggregates.

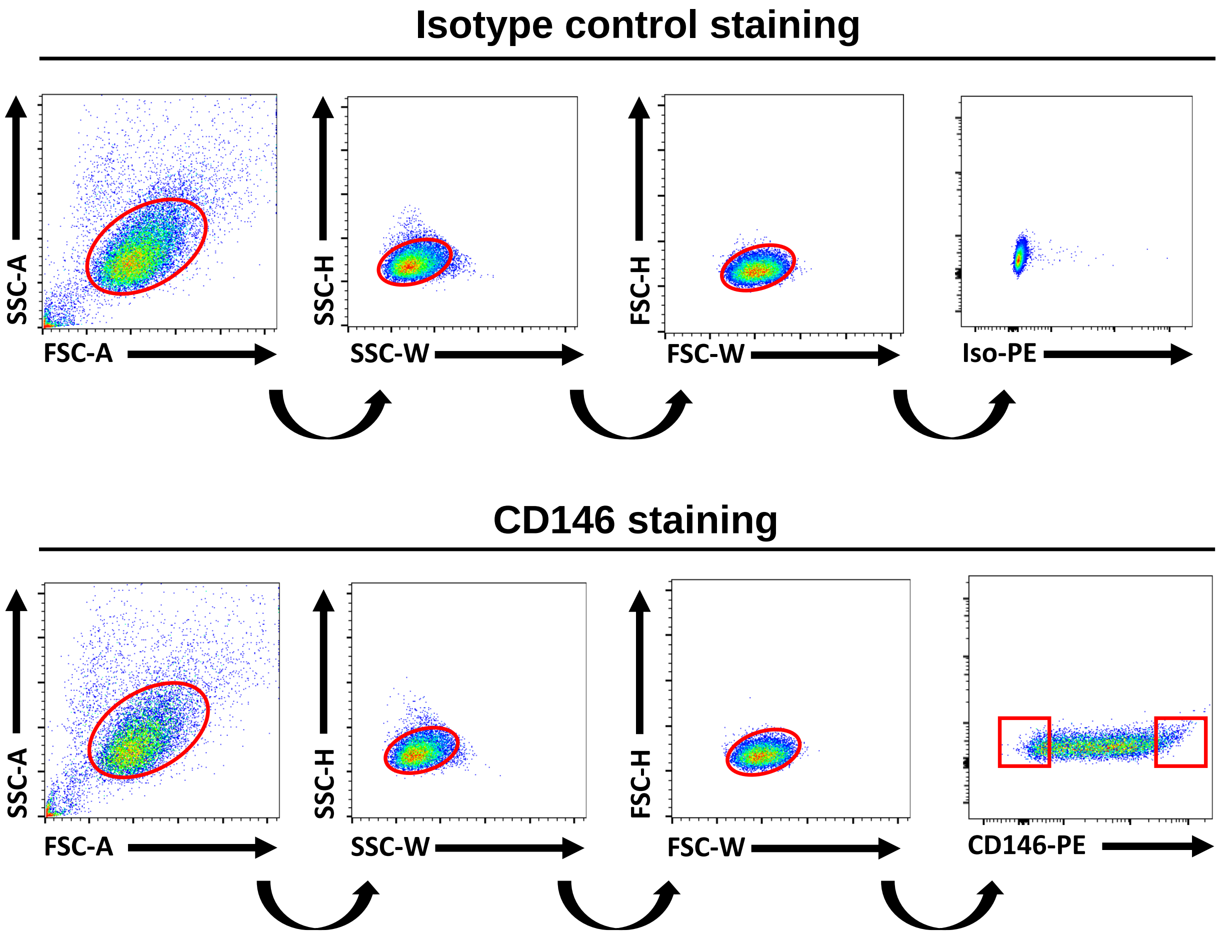

Supplementary figure S2: Gating strategy used for flow-cytometry analysis and sorting. The upper panels represent the CD146 isotype staining. The lower panels represent the gating strategy using the CD146 antibody.
